# Evaluating repellence properties of a catnip essential oil-based mosquito repellent using the human landing catch method in Eastern Uganda

**DOI:** 10.1038/s41598-026-42618-5

**Published:** 2026-03-14

**Authors:** Charles Batume, Isaac Ssegujja, Grace Kongai, Brenda Ayebare, Richard A. Ludlow, Lorenz K. Fuchs, Sarah Mutaka Logose, John Ssebaale, Peter Randerson, Ivan M. Mukisa, John A. Pickett, Simon Scofield

**Affiliations:** 1 Department of Entomology, Uganda Virus Research Institute (UVRI), Entebbe, Uganda; 2 Uganda Industrial Research Institute, Kampala, Uganda; 3 School of Biosciences, Cardiff University, cardiff, UK; 4 School of Chemistry, Cardiff University, cardiff, UK; 5 CEMPOP Uganda, Ltd, Kampala, Uganda; 6 Department of Food Technology and Nutrition, Makerere University, Kampala, Uganda

**Keywords:** Insect repellent, Nepetalactone, Catnip, Catmint, DEET, Mosquito, Human landing catch method, Diseases, Ecology, Ecology, Zoology

## Abstract

Mosquitoes (Diperta: Culidae) act as vectors for several diseases, including malaria, dengue fever and yellow fever. Mosquito repellents represent one of the primary measures used to reduce the risk of these diseases in humans by reducing mosquito landing and biting events. Nepetalactone, a natural insect repellent primarily found in the essential oil of catnip (Lamiaceae; *Nepeta cataria*), has established mosquito repellence properties, but has not been widely exploited as a mosquito repellent in malaria-endemic regions such as Sub-Saharan Africa. Here, we evaluated the potential of a locally produced lotion containing catnip essential oil (comprising > 92% nepetalactone) for use as a mosquito repellent in Eastern Uganda. Using the human landing catch method in field trials, we analysed the effectiveness of a lotion containing 2% or 6% catnip oil at repelling mosquitoes compared to a lotion lacking catnip essential oil as a negative control and to a commercial repellent containing 15% DEET as a positive control. We found that lotions containing both concentrations of catnip essential oil were highly effective at preventing mosquito landing, with 6% catnip oil performing as well as 15% DEET. Our findings suggest that nepetalactone could be used as a natural, locally sourced and effective alternative to synthetic commercial mosquito repellents, thereby representing a viable import substitution option for protection against mosquito-borne diseases.

**Supplementary Information:**

The online version contains supplementary material available at 10.1038/s41598-026-42618-5.

## Introduction

Mosquitoes (Diptera: Culicidae) act as vectors of pathogens causing diseases such as malaria, dengue fever, filariasis, and West Nile virus^1^, and hence pose a major threat to human health. Protecting against mosquito bites typically involves limiting outdoor activities during periods of high mosquito activity, wearing protective clothing and using insect repellents. Although indoor control measures such as indoor residual sprays and insecticide-treated nets have been implemented in regions such as sub-Saharan Africa, outdoor exposure to mosquitoes during work hours remains a key and rapidly growing challenge, particularly for the mosquito species *Anopheles gambiae sensu stricto* (Giles), *Aedes aegypti* (Linnaeus) and *Culex quinquefasciatus* (Say). Hence, there is a need for an effective, affordable and user-friendly solution to prevent mosquito bites and reduce the incidence of diseases such as malaria.

One of the most effective and commonly used mosquito repellents is the synthetic compound DEET (*N*,* N*-diethyl-3-methylbenzamide), although concerns have been raised about its potential adverse effects, particularly in pregnant and lactating women and young children^2–4^. However, many studies have found that DEET is safe and rapidly cleared from the body, unless accidentally ingested or used for prolonged periods of time, and the US Centers for Disease Control and Prevention (CDC) still recommend DEET for protection against mosquitoes and other insect disease vectors^5–10^. DEET is used in several commercially available insect repellents, including ‘Peaceful Sleep’ which contains 15% DEET and is manufactured in South Africa and is the most common repellent available in Uganda. Nonetheless, the relatively high cost of imported commercial DEET, which range considerably in price from 5 to 50 USD per 100 g, prevents its use by many individuals living in mosquito-prone regions like sub-Saharan Africa.

Previous research has led to the identification of plant essential oils with insect-repellent properties, such as geraniol, citronella and neem oil^11,12^. However, these natural alternatives have limitations including shorter duration of repellence compared to DEET. However, several studies have indicated that nepetalactone, an iridoid monoterpene found in the essential oil of catnip (Lamiaceae: *Nepeta cataria)*, can act as a highly effective mosquito repellent that has comparable repellence properties to DEET^13–20^. These studies have employed a range of methodologies, primarily utilizing in vitro attraction and repellence assays using mosquito-attractive heat packs and blood samples, or in vivo assays utilizing human subjects (shielded from biting) or Y-tube olfactometers, and analysed the effects on several species of mosquito including *Aedes aegypti* and *Anopheles gambiae s.s*. In general, these studies found that nepetalactone concentrations ranging from as low 1% could provide repellence against several mosquito species, with increased concentrations providing increased efficacy and duration of repellence. Furthermore, nepetalactone has been shown to have highly effective repellence properties against a range of other arthropods, including ixodid ticks and red poultry mites^20,21^, bed-bugs^22,23^, dust mites^24^ and stable flies^25^. Safety assessments, including dermal, oral and inhalation toxicity tests, have deemed *N. cataria* essential oil safe for human use, as confirmed by the United States Environmental Protection Agency^26^, though it has not been as extensively tested as DEET. Additionally, it has been shown that catnip oil did not cause irritation when applied to human skin at a concentration of 25%^27^, further demonstrating its suitability as a topical insect repellent.

Originally developed by Kerr (1933)^28^, the Human Landing Catch (HLC) method is considered to be the gold standard when evaluating insect attraction/repellence in the field and involves the human subject presenting exposed flesh (typically the lower leg) and collecting any mosquitoes that land using an aspirator, followed by species identification and quantification. The HLC method has been used previously to evaluate the efficacy of mosquito repellents in the field. For example, Colucci et al., (2018)^29^ used the HLC and arm-in-cage methods to study the protection times for *para*-menthane-*3*,*8*-diol and DEET. An additional study used the HLC method and the biting method (that allowed landing mosquitoes to feed) to evaluate the protective efficacy of the volatile pyrethroid spatial repellent (VPSR) transfluthrin^30^. This study showed that HLC and the biting method gave similar estimations of protective efficacy, but HLC has the advantage considering the difficulties associated with quantifying biting events in a field setting. A follow-up study using mathematical modelling suggested that the HLC method tended to underestimate the reduction in landing of susceptible mosquitoes in response to pyrethroid repellents, while sometimes overestimating the reduction in landing of knockdown-resistance mosquitoes, such as those exhibiting resistance to pyrethroids^31^. Thus, the HLC method is an established methodology for conducting repellence trials in the field and is therefore suitable for studying the effect of our topical catnip-oil based repellent.

Our previous work used a Y-tube olfactometer^16^ to conduct repellence assays to evaluate the repellence properties of essential oil from a *Nepeta cataria* chemotype (Chemotype A)^20^, which predominantly contains the 4a*S*,7*S*,7a*R* isomer of nepetalactone at a concentration of > 95%. We found that concentrations as low as 2% provided effective repellence against *Aedes aegypti*, whether applied in a simple carrier oil (e.g. olive oil) or as a constituent of a lotion that can be applied to the skin, which provided up to 4 h of repellence.

In this study, we sought to determine if a catnip essential oil-based repellent lotion, produced using essential oil extracted from locally cultivated *Nepeta cataria* plants in Eastern Uganda, could offer protection from mosquitoes during the evening hours (18:00–22:00), when both humans and mosquitoes are active. We developed the repellent lotion using 2% catnip essential oil, representing the low efficacy threshold established in our previous work, and 6% catnip essential oil, which was more effective yet still represented a sufficiently low concentration to enable cost-effective and sustainable lotion production. We used the human landing catch method to evaluate the effectiveness of the repellent lotions at repelling mosquitoes in two independent field trials, each undertaken at two different locations, together with a placebo trial using a lotion that lacked catnip essential oil. We found that both concentrations provided highly effective repellence, with 6% lotion being as effective as commercial preparations of 15% DEET (branded Peaceful Sleep), while the placebo trial showed no significant repellence. This investigation shows that locally sourced nepetalactone makes a promising alternative to chemical-based repellents like DEET and could form the basis for the development of effective and accessible mosquito repellents for populations in mosquito-borne disease endemic regions such as Eastern Uganda.

## Materials and methods

The study was conducted in compliance with the protocol for Good Clinical Practice and the applicable regulatory requirements in Uganda. The project was approved by the Cardiff University School of Biosciences Research Ethics Committee and the Research Ethics Committee of the CURE Children’s Hospital of Uganda (CCHU-REC), and all research was performed in accordance with the relevant guidelines and regulations. All plants were cultivated in compliance with Ugandan and UK regulatory requirements. All participants in the field trials were local residents of the Kamonkoli Subcounty or Mugiti Subcounty of the Budaka district in Eastern Uganda. All participants were given detailed information about the trial, including any risks, and signed informed consent forms prior to participation (note that if the participant could not read then the consent form was read to them, and translated into the local language if necessary, before signing), and were given a remittance for their time. Potential participants were screened for malaria prior to the field trials and any individuals who tested positive were excluded from the study and given free treatment for malaria. All participants were tested again for malaria after the conclusion of each day of the trial, but none tested positive.

### Purification and analysis of catnip essential oil and repellent lotion formulation

*Nepeta cataria* ‘Chemotype A’ seeds were obtained from CN Seeds (Cambridgeshire, UK). Essential oil from this *N. cataria* chemotype (Chemotype A) has previously been shown to contain > 95% 4a*S*,7*S*,7a*R* (*S*,*S*,*R*) and 4a*S*,7*S*,7a*S* (*S*,*S*,*S*) isomers of nepetalactone and has significant insect repellent quality that has been shown to be highly effective against several species of mosquito, ticks and mites^16,20^. *N. cataria* plants were cultivated in outdoor plantations in the Budaka district of Eastern Uganda with approximately 12 h of daylight per day. Mature plant material was harvested and dried before being distilled. Catnip essential oil was obtained by steam distillation of dried mature *Nepeta cataria* aerial tissue using Clevenger-style distillation equipment. Hexane was added to the distilled fraction and mixed thoroughly and allowed to settle, and the top oil layer was removed and the hexane allowed to evaporate, leaving pure *N. cataria* essential oil.

Catnip essential oil analysis was conducted according to Batume et al. (2024)^16^. Catnip essential oil samples were diluted by a factor of 100 in hexane (> 99%, Sigma-Aldrich). Samples were injected into a Thermo Trace 1300 gas chromatograph fitted with a Thermo TG-5MS column (Thermo Fisher Scientific; 30 m x 0.25 mm x 0.25 μm) and detected using a Thermo ISQ LT mass spectrometer (Thermo Fisher Scientific). The injection port was operated at 200 °C, into which 1 µL of the sample was injected and loaded onto the column at a 1:5 split ratio, with the column being operated at 1 mL min^− 1^ He carrier gas. The GC oven was run with a ramped temperature profile; initial temperature 50 °C for 2 min, ramp at 5 °C min^− 1^ to 230 °C and held for 12 min. The mass spectrometer was operated with a transfer line temperature of 250 °C, an ion source temperature of 230 °C and a mass scan range of 35–350, with a 2 min solvent delay.

GC–MS data were processed using Chromeleon (Version 7.2 SR4; Thermo Scientific, USA) and the deconvolution and integration of signal peaks was made in AMDIS (NIST, 2014), using a custom retention-indexed mass spectral library as previously described^32^. Compounds scoring > 80% in both forward and reverse fit which also had a retention index match of ± 15 were included in the custom library as putatively identified. The integrated signal of all identified peaks was summed for each sample, and results expressed as relative abundance (%) of each compound within a sample. All GC-MS work was conducted in the Biomolecular Research Hub, School of Biosciences, Cardiff University, UK.

The lotion for assessing the duration of repellence was made at the Uganda Industrial Research Institute, Kampala, Uganda, and contained 2% or 6% catnip oil as the active ingredient, in addition to water, glycerin, emulsifying wax, cetyl alcohol, cetyl stearyl alcohol, shea butter, glycerol monostearate, olive oil, coconut oil, sunflower oil, methyl paraben, propyl paraben and silicone oil. For the plain negative control lotion, catnip oil was omitted from the formulation. For the placebo trial, the same plain lotion formulation was used but contained 2% or 6% locally purchased plant essential oil lacking nepetalactone and instead containing ~ 23.6% 3-carene, 12.9% limonene, 8% citral, 7.3% 4-carene, 1% gamma terpinene and 0.6% beta-myrcene with the remainder comprising mostly oxalic acid esters and tricosanol acetate. Compositional analysis of this essential oil was conducted by the Directorate of Government Analytical Laboratory (DGAL) in Uganda. All plant research was conducted in compliance with international and UK guidelines. No endangered species were used in this research.

### Human landing catch field trial design and procedure

**Fig. 1:**
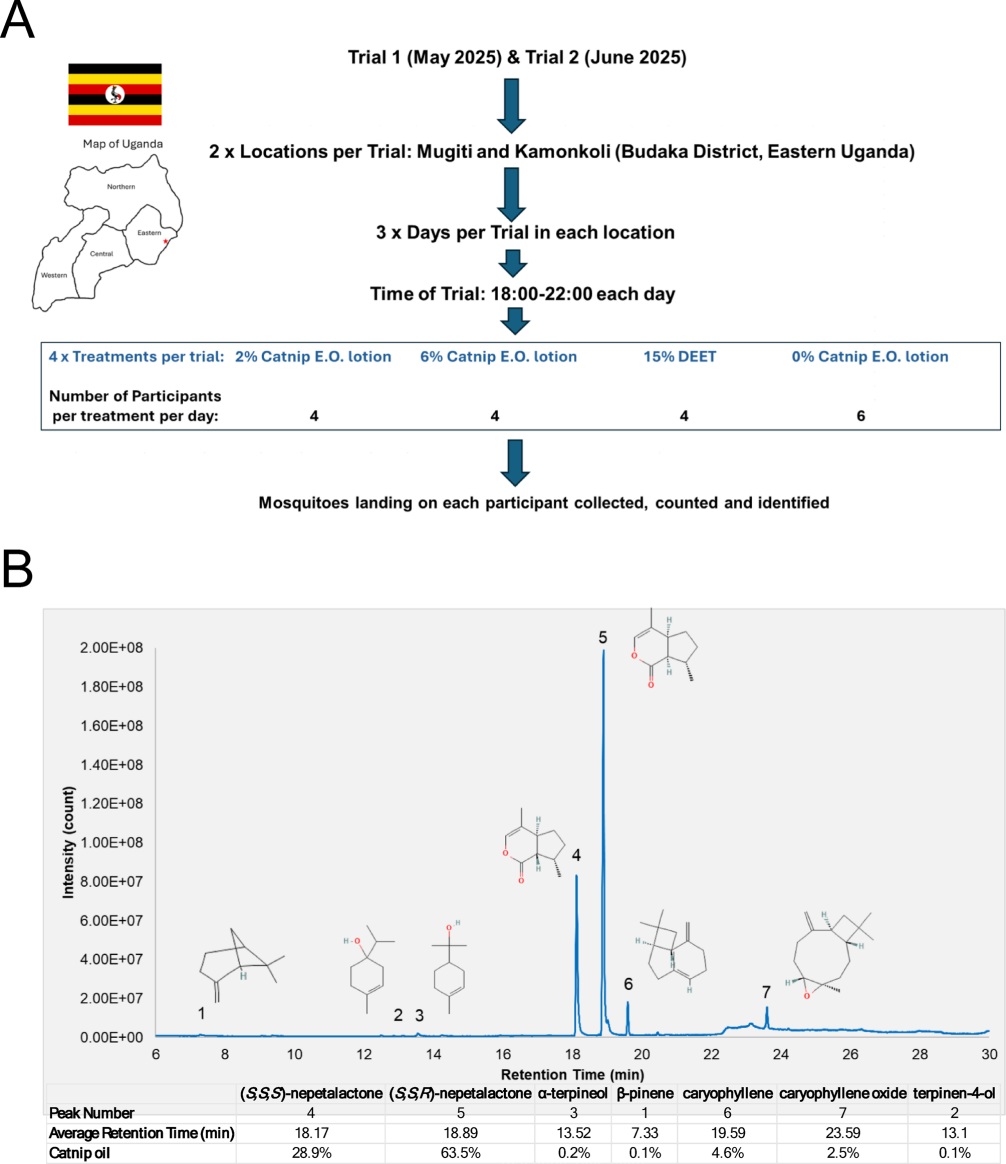
Field trial structure and GC-MS analysis of catnip essential oil composition. **(A)** Schematic representation of Human Landing Catch Field Trial design. Two main trials were conducted in May 2025 and June 2025, each at two neighbouring locations (Mugiti and Kamonkoli; red star on map) over the course of three days. Human participants were randomly assigned to one of four treatment groups (2% catnip oil lotion, 6% catnip oil lotion, 15% DEET or plain lotion control lacking catnip oil). Four participants were randomly assigned to each group on each day, except for the plain lotion group which had six participants per day. Mosquitoes were collected over a four-hour time period each day (18:00–22:00) and were counted and identified. Map of Uganda produced using the GNU image manipulation program (GIMP 2.10.38 revision 1; https://www.gimp.org) **(B)** GC-MS analysis of catnip essential oil obtained by steam distillation of *Nepeta cataria* chemotype A leaf and stem tissue cultivated in outdoor plantations in the Budaka District in Eastern Uganda. Chemical structures of major peaks are shown and compounds are listed in the subtending table together with retention times and relative abundance expressed as a percentage of the catnip oil composition. NIST spectral matches for each compound are shown in Fig. S1.

The methodology used in this study was based on the Human Landing Catch (HLC) method described in Service (1993)^33^ and the well-tested protocol described in Russell et al. (2022)^34^. To evaluate the effectiveness of a locally produced lotion containing nepetalactone as the active mosquito-repelling ingredient, we conducted two field trials using the HLC method, each at two different locations in rural Eastern Uganda (Mugiti and Kamonkoli subcounties of the Budaka District) over the course of 3 days (Fig. 1A) in May and June 2025, and a third smaller-scale placebo trial at a single location (Kamonkoli) using a control lotion containing locally purchased essential oil that did not contain nepetalactone in June 2024. The catnip essential oil used in the two main trials was obtained by steam distillation of leaves and stems of a variety of *Nepeta cataria* designated as Chemotype A^20^ which was cultivated in Eastern Uganda in small plantations under ~ 12 h of daylight per day. GC-MS analysis of the catnip essential oil showed it contained ~ 92.4% nepetalactone (a mixture of *S*,* S*,*R* and *S S*,*S* enantiomers), ~ 4.6% caryophyllene and ~ 2.5%% caryophyllene oxide, and very small amounts of other monoterpenoids (Fig. 1B; Fig. S1). Hence, the oil contained a sufficient concentration of nepetalactone for use in mosquito repellence experiments.

We used the distilled catnip oil to formulate a repellent lotion containing either 2% or 6% catnip oil (see above for full composition). We then evaluated the efficacy of these lotions at repelling mosquitoes using the Human Landing Catch (HLC) method. In each of the main trials there was a total of 18 participants per day per location, who were randomly assigned to one of four treatment groups on each of the three days of the trial. Each treatment group was given a lotion containing 2% catnip essential oil, 6% catnip essential oil or, as a negative control, a lotion lacking any catnip essential oil, but which was otherwise identical in composition to the lotions containing 2% or 6% catnip essential oil. Participants in the positive control group were given a commercial preparation of 15% DEET (branded Peaceful Sleep Insect Repellent Cream). Participants were not told which treatment group they had been assigned to. On each of the three days of the trial, four participants were used to evaluate the effectiveness of each treatment, except for the negative control lotion which was used to treat six participants. The lotions (~ 3 grammes per treatment) were applied to the exposed lower leg (calf and shin) of each participant at the beginning of the 4-hour test period in the evening (18:00 to 22:00), which represented the typical evening hours that participants would spend outdoors undertaking chores or socialising, with the remainder of the body covered with clothing. Participants’ heads and necks were partially covered using a hooded garment, leaving only their faces exposed, and hands were covered with gloves. Participants were seated away from houses and livestock, at least 10 m apart from each other, and used aspirators (Standard Mouth Aspirator Model 412) to collect all mosquitoes that landed on the exposed leg and stored them in a collection jar that prevented escape. All participants were given training for collecting mosquitoes using an aspirator for 2-days prior to the 3-day trial period. Participants were unable to count mosquitoes that landed and escaped before being collected due to the inherent difficulty of tabulating such data in field conditions in the dark. Therefore, we only counted mosquitoes that were collected and made the assumption that the proportion of mosquitoes that escaped prior to capture versus those actually captured would have been similar among participants. Trials began at 18:00 (before sunset at ~ 18:45) and continued until 22:00. During periods of darkness, flashlights were turned on when mosquitoes landed to aid with aspiration. During the first main trial in early May 2025 (May 3rd to May 5th) the average temperature varied from ~ 24 °C at 18:00 to 20.5 °C at 20:00 and the humidity during this period was ~ 78%. On Day 2 and 3 (May 4th and 5th) there was rainfall during the daytime, but only on Day 3 did this persist in the trial period until 8pm. During the second main trial in early June 2025 (June 1 st to June 3rd) the average temperature varied from ~ 24 °C at 18:00 to 19.5 °C at 20:00 and the humidity during this period was ~ 50–55%. On Day 1 and 3 (June 1st and 3rd) there was rainfall during the daytime, but it did not persist into the trial period. At the end of the trial period, each participant returned their collected mosquitoes to the field technicians who stored them for counting and for species identification based on morphology and assigned them to one of six genus/species level classifications (*Anopheles gambiae s.s.*, *Anopheles coustani*, *Culex spp*., *Mansonia spp*., *Aedes aegypti* and *Aedes simpsoni*). The sex of the mosquitoes was not recorded.

### User acceptance survey

The user acceptance survey was developed by CEMPOP Uganda Ltd. in collaboration with KTC consultants, Kampala, Uganda. Demographic data of participants were collected (sex, age, level of education, employment status). Ordinal data on the participants’ experiences with mosquitoes and malaria were collected using a Likert scale, as well as data on awareness of malaria prevention measures and the use of mosquito repellents. Ordinal data using a Likert scale were then collected for the 6% catnip essential oil repellent lotion for effectiveness, appearance, smell, ease of application, feel on the skin, general impression and willingness to purchase if it was commercially available. In total, 119 participants completed the user acceptance survey, comprising field trial participants and their family members.

### Data analysis and statistical tests

All data in the form of number of mosquito landings per participant were collected and processed in Microsoft Excel. The number of mosquitoes collected per participant during each one hour timeslot (18:00–19:00, 19:00–20:00, 20:00–21:00, and 21:00–22:00) were pooled to provide a total number of mosquitoes collected per participant (4 participants for 2% catnip, 6% catnip and 15% DEET repellents, and 6 participants for 0% catnip control lotion) on each day at each location for each of the two trials (i.e. a total of 12 participants for 2% catnip, 6% catnip and 15% DEET repellents, and 18 participants for 0% catnip control lotion per location per trial). Additionally, numbers of mosquitoes recorded in each timeslot for each treatment were used for the repellent duration analysis. The median and mean number of mosquitoes collected per participant for each treatment (pooled and per timeslot) in each location for each trial was calculated along with the associated interquartile range (IQR) and standard deviation (SD), respectively. Statistical significance of the difference in numbers of mosquitoes landing per person per treatment (pooled and per timeslot) was determined using a Kruskal-Wallis test followed by post-hoc Dunn’s test with Bonferroni correction with the online tool: https://www.statskingdom.com/kruskal-wallis-calculator.html. Most data were shown not to be normally distributed, so this choice of a non-parametric test was deemed the most suitable. The same statistical test was used for comparing mosquito species distributions in each treatment. Statistical significance for each pairwise comparison between treatments in each trial and location is given as a p-value (* *p* < 0.05, ** *p* < 0.01, ***p < 0.001). All raw data and processed data (including p-values) are shown in the Supplementary Data spreadsheet.

## Results

### Field trial 1

**Fig. 2:**
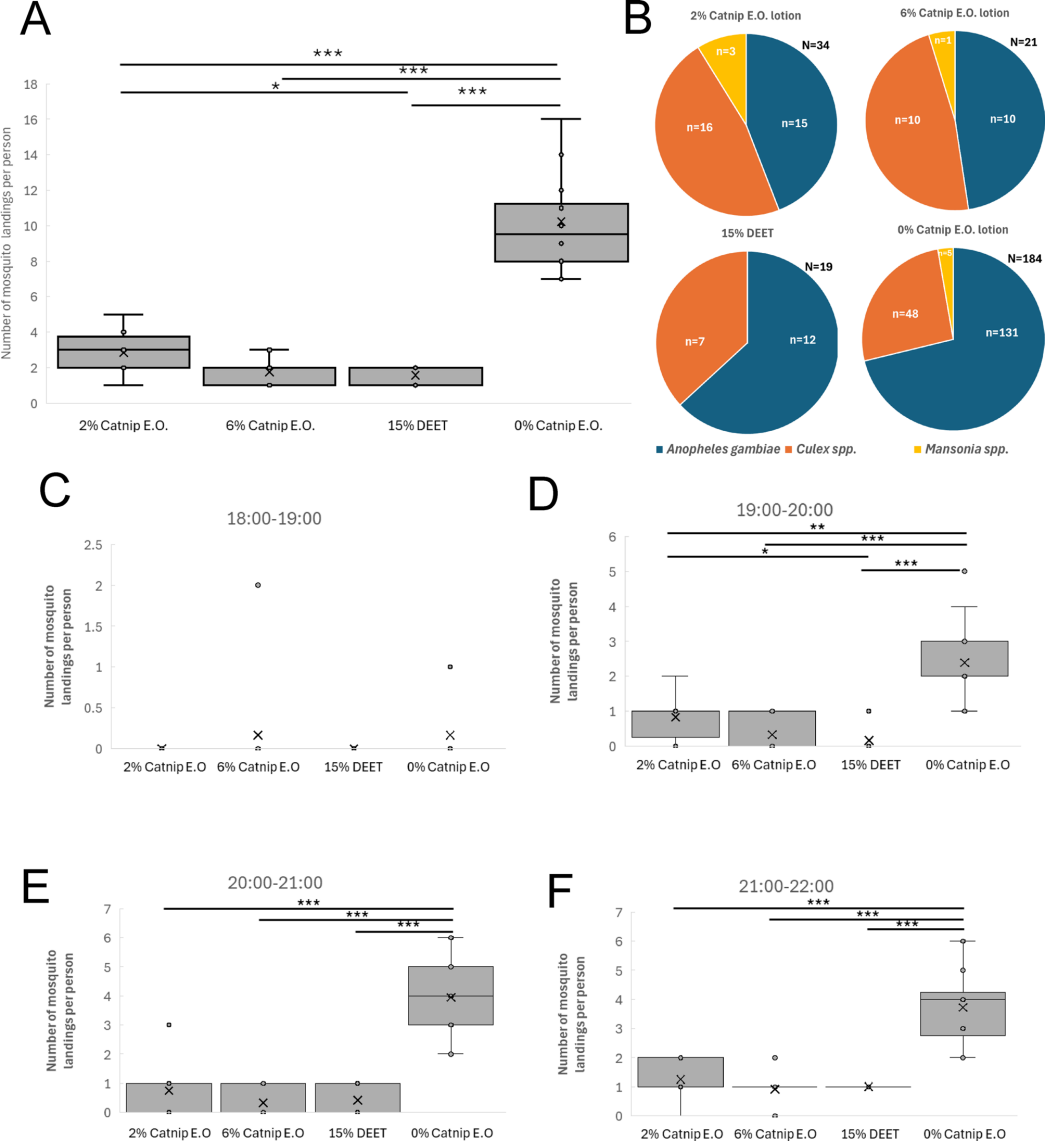
Results of Field Trial 1 in Mugiti (May 2025). **(A)** Human Landing Catch data displaying the total number of mosquito landing events per participant during the four-hour sampling period (18:00 to 22:00) from Field Trial 1 conducted in Mugiti. The data from each of the three days of the trial have been combined. **(B)** Species distribution for mosquito landing events and total number of mosquito landing events per treatment in Field Trial 1 conducted in Mugiti. **(C-F)** Human Landing Catch data displaying the number of mosquito landing events per participant in each one-hour timeslot from Field Trial 1 in Mugiti. The data from each of the three days of the trial have been combined. The box and whisker plots show the median value (black horizontal line), mean value (X), upper and lower quartiles (grey box area), minimum and maximum values excluding outliers (whiskers) and outlier values (individual circular datapoints outside the whiskers). Asterisks indicate statistical significance in a non-parametric Kruskal-Wallis test with post-hoc Dunn’s test (* p < = 0.05, ** p < = 0.01, *** p < = 0.001). All data are shown in the Supplementary Data spreadsheet.

In the first field trial in Mugiti in May 2025 (Fig. 2A), the median number of mosquito landing events per participant treated with the 0% catnip essential oil control lotion on each day of the trial period (3 days; 18:00–22:00 each day) was 9.5 with an interquartile range (IQR) of 3.25 with a mean of 10.22 and standard deviation (SD) of 2.53, while for those treated with 15% DEET the median fell to 2 (IQR = 1) with a mean of 1.58 (SD = 0.51). For those treated with the 2% catnip essential oil lotion, the median number of landing events per participant was 3 (IQR = 1) with a mean of 2.83 (SD = 1.11) while for 6% catnip essential oil the median was 2 (IQR = 1) with a mean of 1.75 (SD = 0.75). The number of landing events in all repellent treatments (2% action, 6% catnip, 15% DEET) was significantly lower compared to the 0% catnip control lotion using the non-parametric Kruskal-Wallis test with post-hoc Dunn’s test (*p* < 0.001; see Methods). The number of landings in the 2% catnip treatment was also significantly higher compared to 15% DEET (*p* < 0.05), suggesting that it was not as effective. Using the median values, the percentage repellence was 68% for 2% catnip, 79% for 6% catnip and 79% for 15% DEET compared to the 0% catnip control lotion (Supplementary Data). The primary species that landed on participants in the control treatment group was *Anopheles gambiae s.s.* followed by *Culex* spp., with only a few *Mansonia* spp. collected (Fig. 2B). For catnip repellent treatments, there was a more equal distribution of *Anopheles gambiae* and *Culex* spp. Statistical analysis (Kruskal-Wallis with post-hoc Dunn’s test) revealed that the distribution of mosquito species in the 2% catnip, 6% catnip and 15% DEET treatments differed significantly from the 0% catnip control, with fewer *Anopheles gambiae s.s.* and more *Culex spp.* collected.

To evaluate the duration of repellence provided by the different repellents, we compared the effects of the different treatments during each one-hour timeslot throughout the four-hour testing window. There was no significant difference between repellents and the 0% catnip essential oil control lotion during the 18:00–19:00 timeslot due to the low number of landings (Fig. 2C), which was presumably due to the presence of daylight during most of this period. When comparing the different repellents in the 19:00–20:00, 20:00–21:00 and 21:00–22:00 timeslots, all showed a significant reduction in landing events compared to the 0% catnip essential oil control lotion, and landings in the 2% catnip treatment were significantly higher compared to DEET in the 19:00–20:00 timeslot only (Fig. 2D-F). Hence, the 2% catnip, 6% catnip and 15% DEET treatments all provided significant repellence compared to the 0% catnip control lotion from 19:00 to 22:00.

**Fig. 3:**
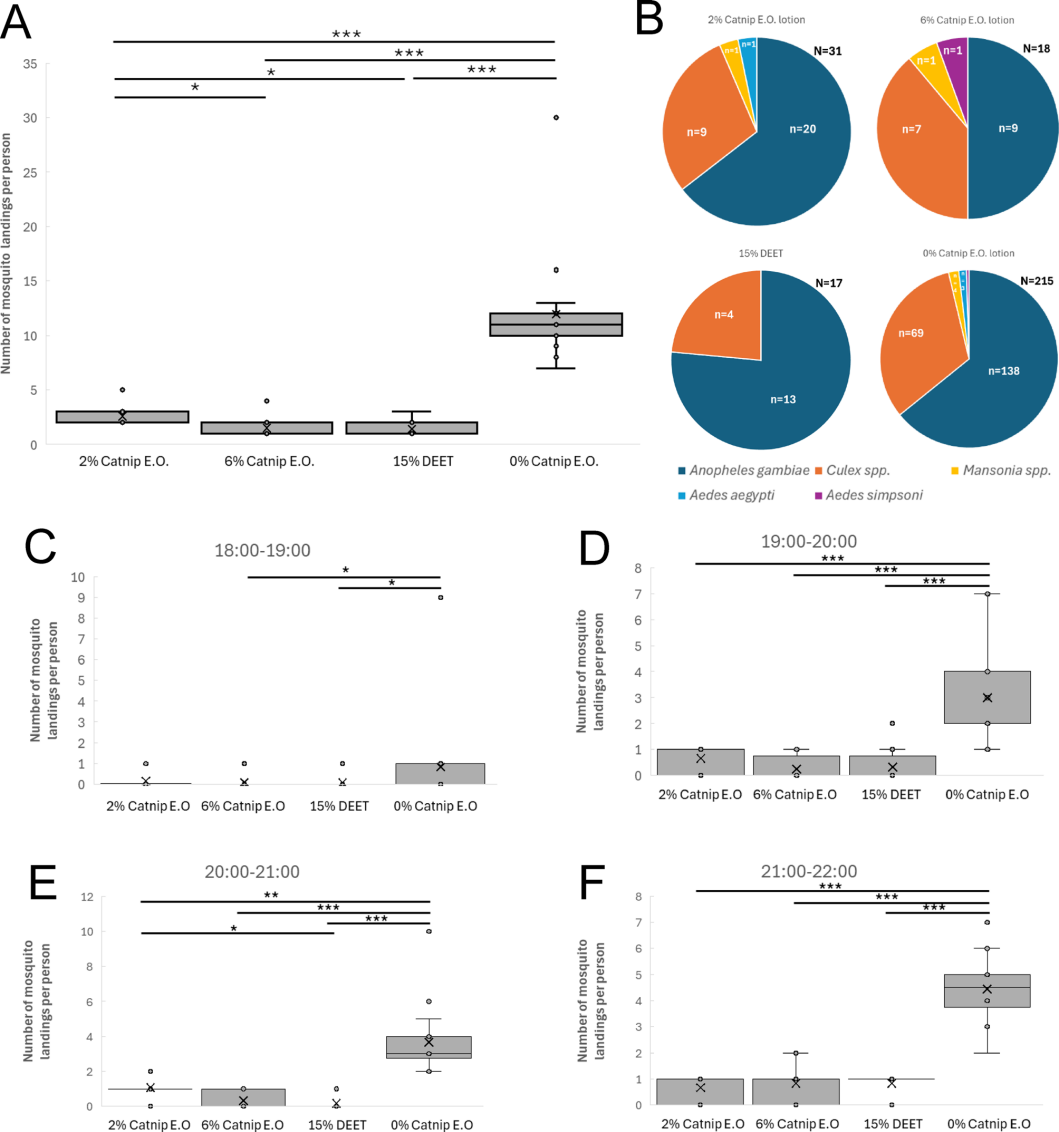
Results of Field Trial 1 in Kamonkoli (May 2025). **(A)** Human Landing Catch data displaying the total number of mosquito landing events per participant during the four-hour sampling period (18:00 to 22:00) from Field Trial 1 conducted in Kamonkoli. The data from each of the three days of the trial have been combined. **(B)** Species distribution for mosquito landing events and total number of mosquito landing events per treatment in Field Trial 1 conducted in Kamonkoli. **(C-F)** Human Landing Catch data displaying the number of mosquito landing events per participant in each one-hour timeslot from Field Trial 1 in Kamonkoli. The data from each of the three days of the trial have been combined. The box and whisker plots show the median value (black horizontal line), mean value (X), upper and lower quartiles (grey box area), minimum and maximum values excluding outliers (whiskers) and outlier values (individual circular datapoints outside the whiskers). Asterisks indicate statistical significance in a non-parametric Kruskal-Wallis test with post-hoc Dunn’s test (* p < = 0.05, ** p < = 0.01, *** p < = 0.001). All data are shown in the Supplementary Data spreadsheet.

Similar results were obtained in the first field trial in Kamonkoli in May 2025 (Fig. 3A). Participants treated with 0% catnip control lotion recorded a median of 11 landing events (IQR = 2) with a mean of 11.94 (SD = 4.92), while those treated with DEET recorded a median of 1 (IQR = 1) with a mean of 1.42 (SD = 0.67). For participants treated with 2% catnip essential oil lotion, the median number of landing events was 2 (IQR = 1) with a mean of 2.58 (SD = 0.90) while for 6% catnip the median was 1 (IQR = 1) and the mean was 1.50 (SD = 0.90). The number of landing events per person for all treatments with repellents was significantly lower compared to the 0% catnip control lotion (*p* < 0.001). The 2% catnip lotion was significantly different from both DEET (*p* < 0.05) and 6% catnip (*p* < 0.05), again suggesting it was less effective. Using the median values, the percentage repellence was 82% for 2% catnip, 91% for 6% catnip and 91% for 15% DEET compared to the 0% catnip control lotion (Supplementary Data). The species distribution was similar to the trial in Mugiti, but with the addition of a few *Aedes* spp. mosquitoes (Fig. 3B). Statistical revealed that the distribution of mosquito species in the 2% catnip, 6% catnip and 15% DEET treatments differed significantly from the 0% catnip control for *Anopheles gambiae s.s.* and *Culex spp.*

We then evaluated the duration of repellence in the same way as for the data from Mugiti, and found similar results, with the 2% catnip, 6% catnip and 15% DEET treatments all providing significant repellence compared to the 0% catnip control lotion from 19:00–22:00 (Fig. 3C-F), and a significant increase in landings in the 2% catnip treatment compared to 15% DEET in the 20:00–21:00 timeslot. Therefore, the 2% catnip, 6% catnip and 15% DEET treatments once again provided significant repellence compared to the 0% catnip control lotion from 19:00 to 22:00, and also a significant reduction in landings between the 6% catnip and 15% DEET treatments and the 0% catnip control during the 18:00–19:00 timeslot in this location.

### Field trial 2

**Fig. 4:**
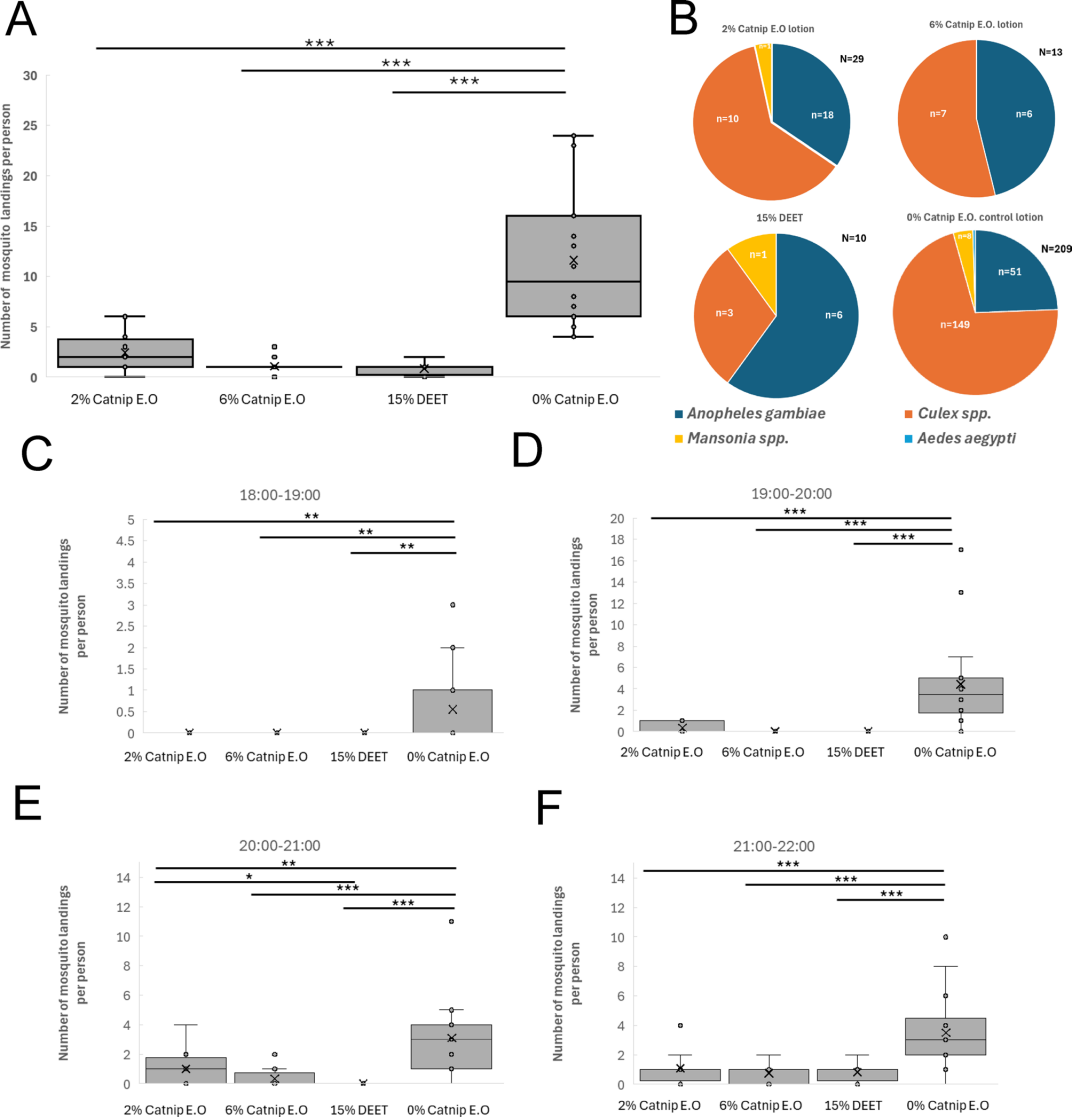
Results of Field Trial 2 in Mugiti (June 2025). **(A)** Human Landing Catch data displaying the total number of mosquito landing events per participant during the four-hour sampling period (18:00 to 22:00) from Field Trial 2 conducted in Mugiti. The data from each of the three days of the trial have been combined. **(B)** Species distribution for mosquito landing events and total number of mosquito landing events per treatment in Field Trial 2 conducted in Mugiti. **(C-F)** Human Landing Catch data displaying the number of mosquito landing events per participant in each one-hour timeslot from Field Trial 2 in Mugiti. The data from each of the three days of the trial have been combined. The box and whisker plots show the median value (black horizontal line), mean value (X), upper and lower quartiles (grey box area), minimum and maximum values excluding outliers (whiskers) and outlier values (individual circular datapoints outside the whiskers). Asterisks indicate statistical significance in a non-parametric Kruskal-Wallis test with post-hoc Dunn’s test (* p < = 0.05, ** p < = 0.01, *** p < = 0.001). All data are shown in the Supplementary Data spreadsheet.

In the second field trial in Mugiti in June 2025 (Fig. 4A), the median number of mosquito landing events per participant per day for those treated with 0% catnip essential oil control lotion was 9.5 (IQR = 10) with a mean of 11.61 (SD = 6.78), while for those treated with DEET the median number fell to 1 (IQR = 0.75) with a mean of 0.83 (SD = 0.58). For 2% catnip essential oil, the median number of landing events per participant was 2 (IQR = 2.75) with a mean of 2.42 (SD = 1.98) while for 6% catnip oil the median number was 1 (IQR = 0) with a mean of 1.08 (SD = 0.79). The number of landing events for all treatments with repellents was significantly lower compared to the 0% catnip essential control oil lotion (*p* < 0.001). Using the median values, the percentage repellence was 79% for 2% catnip, 89% for 6% catnip and 89% for 15% DEET compared to the 0% catnip control lotion (Supplementary Data). The primary mosquito genus/species that landed on participants treated with the control lotion was *Culex spp*. followed by *Anopheles gambiae s.s*, with a few *Mansonia* spp. and one *Aedes aegypti*. specimen collected (Fig. 4B). *Culex spp*. was also the dominant genus of mosquito recorded for the 2% and 6% catnip essential oil lotion treatments, while for 15% DEET, *Anopheles gambiae s.s* was the most prevalent. Statistical analysis revealed that the distribution of mosquito species in the 2% catnip, 6% catnip and 15% DEET treatments differed significantly from the 0% catnip control for *Anopheles gambiae s.s.* and *Culex* spp.

We then evaluated the duration of repellence in the same way as for the data from Field Trial 1, and found similar results, with the 2% catnip, 6% catnip and 15% DEET treatments all providing significant repellence compared to the 0% catnip control lotion from 18:00–22:00 (Fig. 4C-F), and a significant increase in landings in the 2% catnip treatment compared to 15% DEET in the 20:00–21:00 timeslot. Therefore, the 2% catnip, 6% catnip and 15% DEET treatments once again provided significant repellence compared to the 0% catnip control lotion for the duration of the trial.

**Fig. 5:**
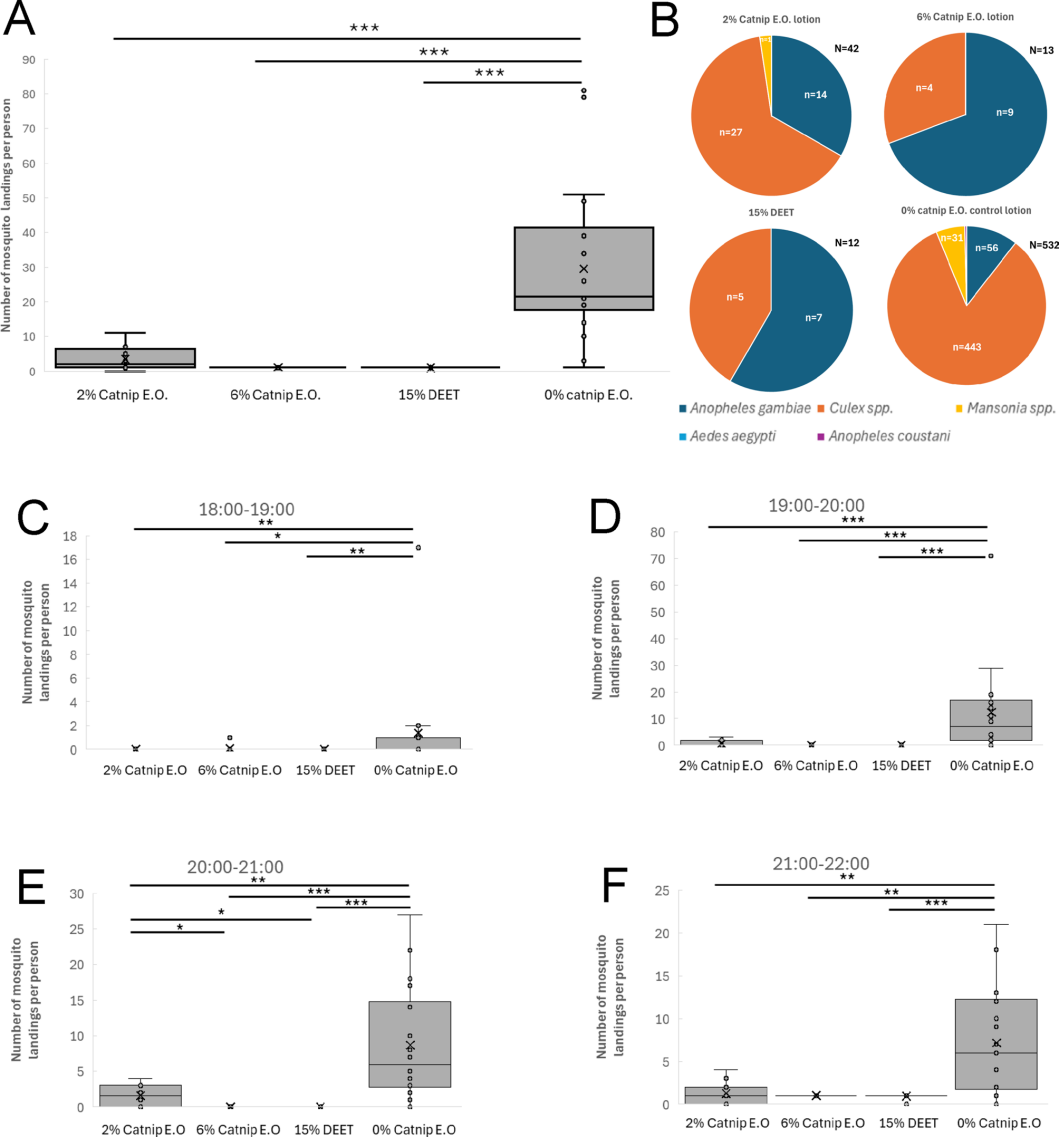
Results of Field Trial 2 in Kamonkoli (June 2025). **(A)** Human Landing Catch data displaying the total number of mosquito landing events per participant during the four-hour sampling period (18:00 to 22:00) from Field Trial 2 conducted in Kamonkoli. The data from each of the three days of the trial have been combined. **(B)** Species distribution for mosquito landing events and total number of mosquito landing events per treatment in Field Trial 2 conducted in Kamonkoli. **(C-F)** Human Landing Catch data displaying the number of mosquito landing events per participant in each one-hour timeslot from Field Trial 2 in Kamonkoli. The data from each of the three days of the trial have been combined. The box and whisker plots show the median value (black horizontal line), mean value (X), upper and lower quartiles (grey box area), minimum and maximum values excluding outliers (whiskers) and outlier values (individual circular datapoints outside the whiskers). Asterisks indicate statistical significance in a non-parametric Kruskal-Wallis test with post-hoc Dunn’s test (* p < = 0.05, ** p < = 0.01, *** p < = 0.001). All data are shown in the Supplementary Data spreadsheet.

Similar results were obtained from the second trial in Kamonkoli in June 2025 (Fig. 5A). Participants treated with 0% catnip control lotion recorded a median number of landing events per participant per day of 21.5 (IQR = 23.75) with a mean of 29.56 (SD = 22.69), while for those treated with 15% DEET the number fell to a median of 1 (IQR = 0) and mean of 1 (SD = 0). For participants treated with 2% catnip lotion, the median number of landing events was 2 (IQR = 5.5) with a mean of 3.5 (SD = 3.32), while for 6% catnip oil the median was 1 (IQR = 0) with a mean of 1.08 (SD = 0.29). The number of landing events for all treatments with repellents was significantly lower compared to the 0% catnip control lotion (*p* < 0.001). Using the median values, the percentage repellence was 91% for 2% catnip, 95% for 6% catnip and 95% for 15% DEET compared to the 0% catnip control lotion (Supplementary Data). The primary mosquito genus/species that landed on participants treated with the 0% catnip control lotion was *Culex spp*., followed by *Anopheles gambiae s.s*, and *Mansonia spp*., with two *Anopheles coustani* specimens collected (Fig. 5B). *Culex spp.* was also the dominant genus of mosquito recorded for the 2% catnip essential oil lotion treatments, while for 6% catnip and 15% DEET treatments, *Anopheles gambiae s.s* was the most prevalent. Statistical analysis revealed that the distribution of mosquito species in the 2% catnip, 6% catnip and 15% DEET treatments differed significantly from the 0% catnip control for *Anopheles gambiae s.s. Culex spp*. and *Mansonia spp.*

We then evaluated the duration of repellence in the same way as for the data from Mugiti, and found similar results, with the 2% catnip, 6% catnip and 15% DEET treatments all providing significant repellence compared to the 0% catnip control lotion from 18:00–22:00 (Fig. 5C-F), and a significant increase in landings in the 2% catnip treatment compared to 6% catnip and 15% DEET in the 20:00–21:00 timeslot. Therefore, the 2% catnip, 6% catnip and 15% DEET treatments once again provided significant repellence compared to the 0% catnip control lotion for the duration of the trial.

### Placebo trial

**Fig. 6:**
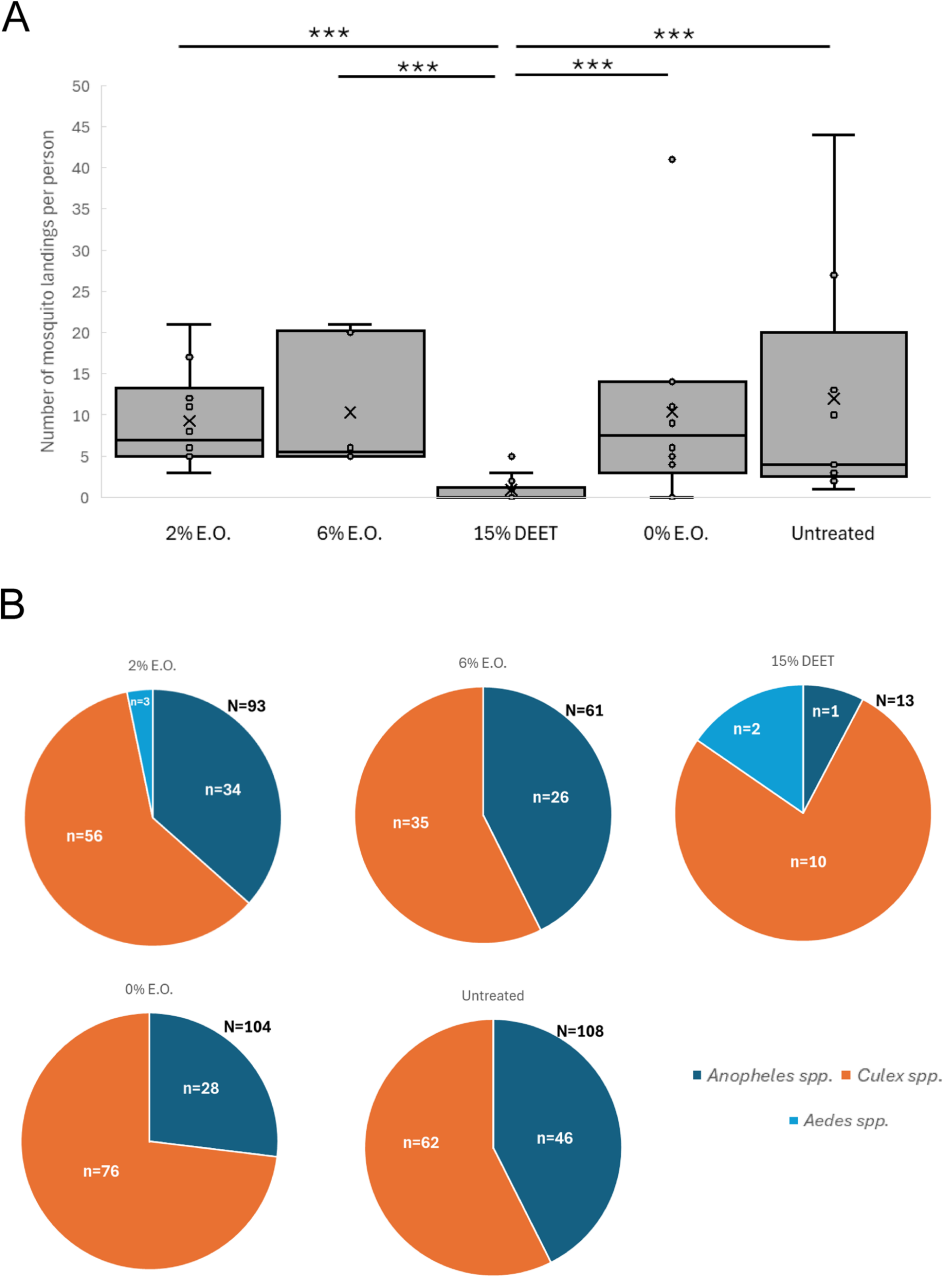
Results of Placebo Trial (June 2024) conducted in Kamonkoli. **(A)** Human Landing catch data from Placebo trial conducted in Kamonkoli in June 2024. 2% E.O. and 6% E.O. are placebo treatments with essential oil lacking nepetalactone. **(B)** Species distribution for mosquito landing events and total number of mosquito landing events per treatment for Placebo Trial conducted in Kamonkoli. Data in A are shown as box and whisker plots displaying the number of mosquito landing events per participant during the four-hour sampling period (18:00 to 22:00). The data from each of the three days of each trial have been combined. The box and whisker plots show the median value (black horizontal line), mean value (X), upper and lower quartiles (grey box area), minimum and maximum values excluding outliers (whiskers) and outlier values (individual circular datapoints outside the whiskers). Asterisks indicate statistical significance in a non-parametric Kruskal-Wallis Test with post-hoc Dunn’s test (* p < = 0.05, ** p < = 0.01, *** p < = 0.001). Note that numbers of participants in each group were not equal (see main text).

To eliminate the possibility of any participant bias in our study we also conducted a smaller-scale placebo trial in Kamonkoli (June 2024) where the repellent placebo lotion was formulated with a plant essential oil lacking nepetalactone, which comprised predominantly 3-carene, 4-carene, D-limonene and citral (Fig. 6; see Methods). Participants receiving the lotions containing 2% and 6% of this essential oil were not informed that the repellent lotion lacked nepetalactone as the active ingredient. Here we also compared the number of mosquito landings for participants that applied no lotion, in addition to the 0% essential lotion, 15% DEET and 2% and 6% essential oil lotions. As shown in Fig. 6A, over the course of the trial, participants with no lotion recorded a median number of landing events per person per day of 4 (IQR = 17.5) with a mean of 12 (SD = 14.51), while for the 0% oil lotion the median was 7.5 (IQR = 11) and the mean was 10.4 (SD = 11.88). For 2% oil the median was 7 (IQR = 8.25) with a mean of 9.3 (SD = 5.91) per participant and for 6% the median was 5.5 (IQR = 15.25) and the mean was 10.3 (SD = 7.89). As expected, 15% DEET had a strong repellent effect with median of 0 (IQR = 1.25) and a mean of 0.92 (SD = 1.49) mosquito landings per participant. The number of landing events for 15% DEET was significantly lower compared to all other treatments (*p* > 0.001), but there was no statistical significance between any other treatments. These results show that the exclusion of catnip oil from the repellent lotion completely negated its effectiveness as a repellent as determined by the human landing catch assay. One point to consider is that the number of participants in this placebo trial assigned to each treatment group varied on different days of the trial. There were 9 participants in total over the 3 days for the no lotion group, 10 for 0% oil lotion, 14 for 15% DEET, 10 for 2% oil and only 6 for 6% oil. The primary mosquito genus/species for all treatments was *Culex spp*. followed by *Anopheles gambiae s.s*, with a few *Aedes aegypti* mosquitoes collected in the 2% essential oil and 15% DEET treatments (Fig. 6B). Statistical analysis revealed that the distribution of mosquito species in the 15% DEET treatment differed significantly from all other treatments for *Anopheles gambiae s.s.* and *Culex* spp., and between 2% oil and all other treatments for *Aedes spp*.

These results show that the landing events for the placebo lotions did not significantly differ from either the 0% control lotion or no lotion treatment groups, but did differ from 15% DEET, indicating that there is no inherent bias among participants attributable to a belief that they may be using a catnip essential oil-based repellent.

### User acceptance survey

**Fig. 7:**
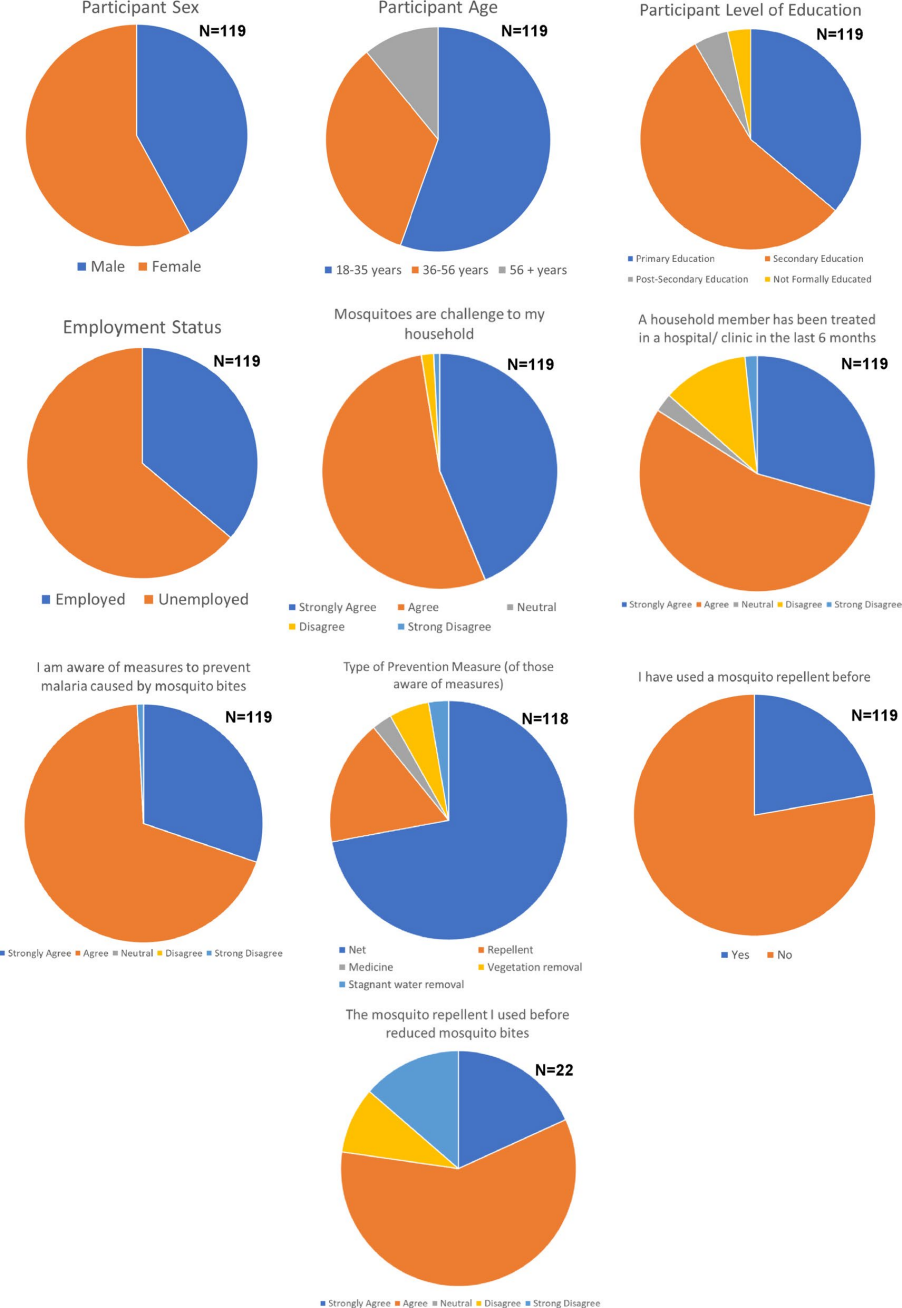
Demographic and malaria knowledge questionnaire responses from the User Acceptance Survey (119 respondents).

After the human landing catch trial in June 2025, samples of our 6% essential catnip oil repellent lotion were given to the HLC trial participants and their family members together with a user acceptance survey to evaluate their perception of the catnip oil-based repellent lotion. A total of 119 participants completed the user acceptance survey following instructions provided by the field technicians. Demographic details of the participants are shown in Fig. 7. The majority of respondents were female (58%) and between 18 and 35 years of age (55%). Most had a secondary level of education (55%) and were not formally employed (64%).

Participants were first asked a series of questions about their experience with mosquitoes and malaria. Questions were phrased as a statement to which participants either strongly agreed, agreed, were neutral, disagreed or strongly disagreed (Likert Scale design; Fig. 7). Data indicated that mosquitoes represent a challenge in the vast majority of households (97%), and 84% reported that a family member had been treated for malaria in a hospital or medical clinic in the last 6 months. Most were aware of prevention measures (99%); primarily the use of bed nets (89%) and to a lesser degree repellents (21%), medication (3%), and removal of vegetation and stagnant water (10%). Only 18% of participants had previously used a different mosquito repellent, and 77% of these found these repellent(s) to be effective at reducing mosquito bites.

**Fig. 8:**
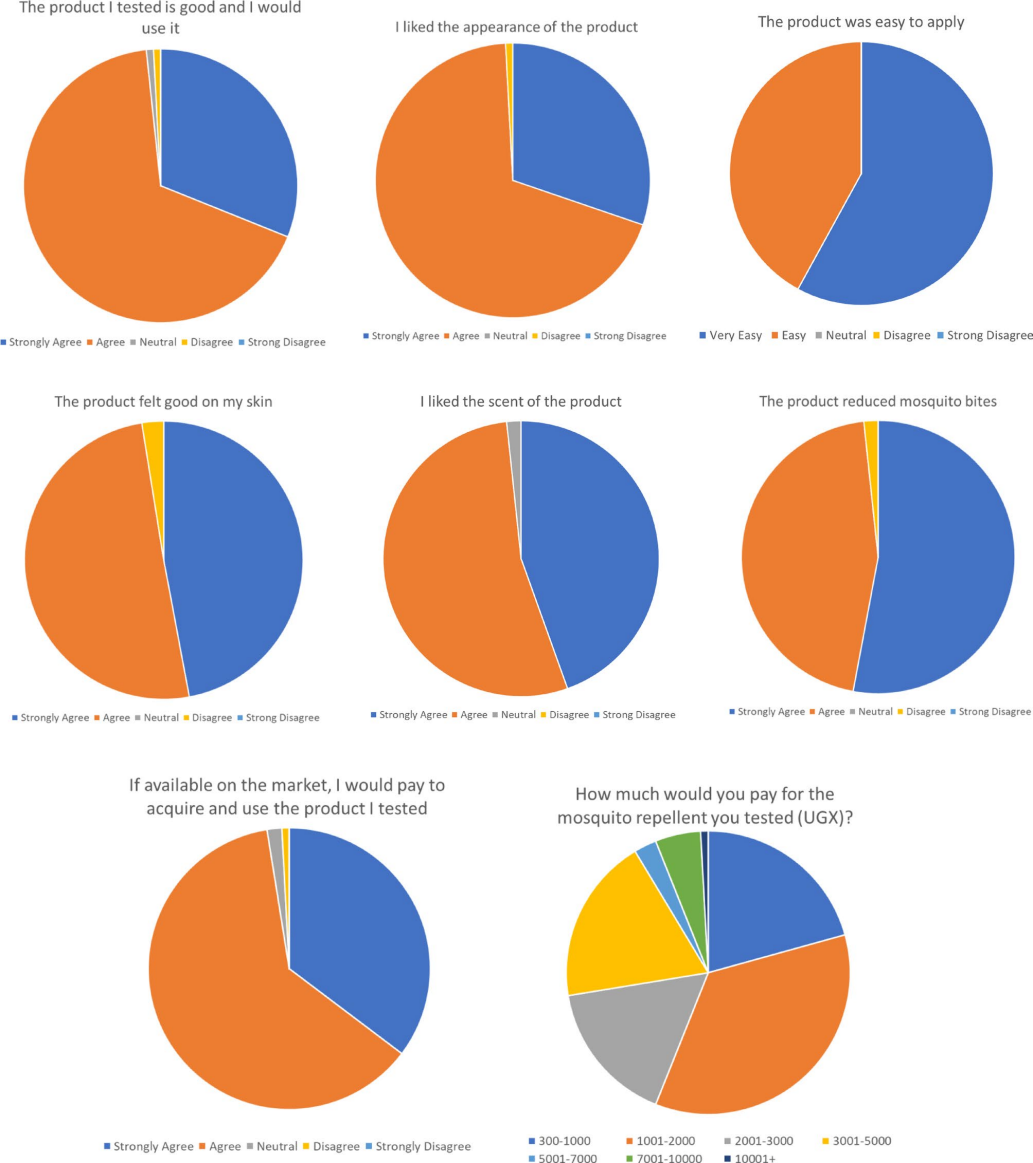
Product evaluation questionnaire responses from the User Acceptance Survey (119 respondents).

A series of questions concerning the participants’ opinion of the 6% catnip essential oil-based repellent were then asked, again framed as statements to which the level of agreement was recorded (Fig. 8). Most participants (98%) thought our repellent was good and would use it, 99% liked the appearance of the product and all participants found it easy to apply. Most participants (97%) said that the product felt good in their skin and 98% liked the scent. The majority agreed that our lotion reduced mosquito bites (98%), and 97% would be willing to purchase the repellent if it was brought to market, with just over half willing to pay between 300 and 2000 UGX for a bottle containing 30 g of repellent, and the remainder being willing to pay between 2000 and 10,000 UGX. Note that this positive evaluation of the effectiveness in repelling mosquitoes was independent of the HLC trials, and was therefore subjective and anecdotal in nature. Overall, the results of the user acceptance survey show that most participants were very happy with all the attributes of our catnip essential oil-based mosquito repellent. They felt that it was effective at reducing mosquito bites and would be willing to purchase it if it was available on the market.

## Discussion

The active compound in catnip essential oil, the iridoid monoterpene nepetalactone, is obtained from the essential oil of the plant *Nepeta cataria*, a member of the Lamiaceae (mint) family. Numerous studies have shown that nepetalactone has significant repellence properties against several hematophagous arthropod species^13–19,20–25^, including mosquitoes which act as vectors for several infectious human pathogens causing diseases, including malaria, in sub-Saharan Africa and elsewhere. For example, Chauhan et al. (2005) found that nepetalactone was effective as repellent against *Aedes aegypti* using human attraction assays and in vitro assays, although it was not as effective as DEET^13^. Likewise, Doraysamy et al. (2012) found that catnip oil concentrations ranging from 10 to 50% provided 62–80% repellence against *Aedes aegypti*, compared to 100% repellence for DEET^14^. By contrast, a study by Reichart et al. (2019) found that 1% catnip oil extract, pure nepetalactone isomers and DEET were equally effective at repelling *Aedes aegypti*, and that 10% catnip essential oil was able to sustain the repellence for 2-4h^15^. In our recent study, Batume et al. (2024) showed that 2% nepetalactone could repel *Aedes aegypti* in the laboratory using a Y-tube olfactometer^16^. The hydrogenated form of nepetalactone has also been shown to have effective mosquito repellence properties. Spero et al. (2008) found that dihydronepetalactone, at 15% in solution, conferred repellence against *Aedes intrudens* (Dyar) for up to 4 h in the field^17^. Furthermore, Feaster et al. (2009) found that dihydronepetalactone effectively repelled mosquitoes at concentrations of 5–10% using human landing assays and in vitro assays^18^. Overall, these studies show that nepetalactone and its derivatives have significant insect repellence properties, although they have yet to be utilised in commercially available insect repellent products. Our data show that a single application of lotion containing 2% or 6% catnip oil (comprising ~ 92% nepetalactone) is highly effective at repelling mosquito landing on human subjects over the course of a 4-hour test period in the evening, when members of rural communities often undertake chores and socialise, compared to the use of a control lotion that lacked catnip oil. This is in agreement with our previous work that showed that a single application of a 2% catnip essential oil lotion was effective at repelling mosquitoes for up to four hours in the laboratory using a Y-tube olfactometer^16^, and other studies that showed mosquito repellence by nepetalactone at a range of different concentrations^13–20^. Furthermore, we found that a lotion containing 6% nepetalactone was as effective as 15% DEET (branded as Peaceful Sleep, a common form of DEET available in rural Uganda) in terms of repellence efficacy. By comparison, our smaller-scale placebo trial showed that the lotion containing 2% or 6% essential oil that lacked nepetalactone showed no detectable repellence. Taken together, the observed repellence when using catnip oil and the lack of repellence using a non-catnip essential oil-based lotion suggests that the repellence observed in our study is attributable to one or more constituents of the catnip essential oil, which included > 92% nepetalactone, ~ 5% caryophyllene, 2.5% caryophyllene oxide and trace amounts of other monoterpenes. Given that the catnip essential oil is mostly composed of nepetalactone, which has established insect repellence properties, we conclude that the repellence we observe is most likely attributable to this compound, though the contribution of these other constituents cannot be excluded. DEET is a broad-spectrum repellent that is highly effective against many species of mosquito, including *Aedes aegypti*, *Culex quinquefasciatus* and *Anopheles gambiae*^35^. The response to DEET is mediated by ordorant receptors (ORs) and Orco co-receptors^36–39^, while nepetalactone has been shown to stimulate olfactory receptor neurons in the basiconic sensilla of the maxillary palps of *Aedes aegypti*^40^, and acts as an agonist for the chemical irritant TRPA1 receptor and in *Aedes aegypti* and the fruit fly *Drosophila melanogaster*^41^. Hence, both act as repellents, but through different mechanisms. Our data indicate that there was a significant difference in the range of mosquitoes captured by participants in the 0% catnip control treatment compared to the DEET, 2% catnip and 6% catnip treatments, suggesting a degree of bias in the action of these repellents against different species. In Field Trial 2, *Culex spp.* mosquitoes accounted for a smaller proportion of mosquitoes in the repellent groups compared to the control. However, there was not a consistent increase or decrease of any particular species in the repellent treatments compared to the control in Field Trial 1. Caution is needed when interpreting these data due to the much smaller sample sizes for mosquitoes collected in the repellent treatments groups compared to the control group, especially considering that a similar difference was observed in the placebo trial. We note that in the Field Trial 2, conducted in June, the proportion of *Culex spp.* mosquitoes collected was far higher than in Field Trial 1, which was conducted in May. This is likely due to the peak abundance of *Culex spp*. between June and August in Africa^42,43^. We note that the low proportion of *Aedes aegypti* collected in our trial may be accounted for by the daily activity pattern of this species, which is most active in the morning and afternoon^44^, whereas our trials were conducted mostly after sunset between 18:00 and 22:00.

The human landing catch (HLC) method is an established methodology for studying mosquito behaviour^28,33,34^ and has been employed in a range of insect attraction/repellence studies, and has been used in a range of different geographical locations to study mosquito landing/biting^45,46^ and to study the effects of mosquito repellents such as DEET, *para*-menthane-3,8-diol (PMD) and nepetalactone^29^, and insecticides such as the volatile pyrethroid spatial repellent transfluthrin^30^. It has been shown to be a highly efficient sampling method, out-performing prevention light trap catches and yeast-generated carbon dioxide-baited light traps in a study conducted in Ethiopia^46^. The same study raised important caveats about the HLC method, such as that it is potentially subject to participant collector bias, and it exposes the participant to increased risk of infectious mosquito bites. To address any bias in our study, participants were assigned to random treatment groups on each day of each trial and were not told which treatment they were testing. One limitation of this is that the distinctive odour of DEET, which acts as both a contact and spatial repellent of mosquitoes^36–39^, would have made it apparent to the participant that they had been given this treatment. The odour of catnip essential oil, however, while notable even at 2% or 6% concentrations, would not have been familiar to the participants and so those given this treatment would be less likely to be able to identify their treatment group. We also conducted a placebo trial in which participants were told that two of the treatment groups contained catnip essential oil as a repellent, whereas in fact this was a plant essential oil that lacked nepetalactone. This placebo oil contained some compounds that have known insect repellence properties, such as limonene, citral and 3-carene^47,48^. However, the relatively lower concentrations of these compounds in the placebo essential oil, compared to the very high concentration of nepetalactone in the catnip essential oil, may account for the lack of repellence effectiveness when diluted to the 2% or 6% essential oil concentrations in the placebo lotions. The lack of any significant reduction in the number of mosquito landings between any group in this placebo trial, except for those treated with 15% DEET, indicates that there is no significant inherent participant bias in our study.

Prior to conducting our trials, all participants were tested for malaria by local medical practitioners and were retested daily throughout the trial. Any putative participants that tested positive for malaria were not eligible to participate in the trial and were given free treatment for malaria. No participants recorded the need for malaria treatment immediately after the trials. A previous study was conducted to investigate the incidence of malaria in HLC trial participants in Kenya. The study found there is no significant increase in malaria incidence in trial participants versus non-participants when proper precautions were taken^49^. Therefore, using the HLC method to evaluate repellence efficacy in the field is an ethical methodology with low inherent risk.

HLC studies have been used extensively to investigate mosquitoes biting humans in the field, including the effect of geographical location-dependent variation in mosquito biting events. In a study conducted in the UK, it was found that geographical location affected the incidence of biting^45^. In our trials, we found similar numbers of landing events in both locations during Trial 1, but more than double the number of landing events in Kamonkoli compared to Mugiti in Field Trial 2. We note that the majority of the increase in landing events in Kamonkoli in Field Trial 2 was limited to the 0% catnip lotion control group, with most participants recording higher numbers of landing events in that group than for the equivalent group in Mugiti, or in either location in Field Trial 1. This could indicate higher than normal numbers of mosquitoes in the location at this time or a higher degree of attractiveness of participants in this group. However, we did not see a concomitant increase in the number of landing events in the other treatment groups, and the randomised design for allocating participants to treatment groups on each day makes it unlikely that human subjects used each day in the 0% catnip lotion group were all super-attractive to mosquitoes compared to those in the other groups. However, there was one participant in particular who was assigned to the plain lotion control group for each of the three days of the trial by chance, recording between 51 and 81 landing events per day, which was well-above the average. It is well known that some individuals are more attractive to mosquitoes than others, with studies indicating the factors such as genetics, skin odours, skin microbiome, pregnancy, *Plasmodium* infection and diet may be involved^50^. In particular, a high emanation of carboxylic acids from human skin has been identified to play a key role in attractiveness, and receptors for these compounds have been identified in mosquitoes^51–53^. This observation highlights the importance of conducting trials at both different locations and at different times, and with as many different human subjects as possible, to capture any variance in mosquito activity or attraction behaviour. Our randomized study design, which involved randomly assigning participants to different treatment groups each day, minimized confounding factors such as inherent variation in attractiveness of the different human participants. Moreover, some participants were involved on more than one daily session, while others participated in only one daily session, thereby increasing the pool of human subjects and the randomized nature of the study. By conducting trials in different locations, over the course of several nights and in different months, we were also able to minimize the effects of geographical location and temporal variance in mosquito activity.

## Conclusions

This study highlights the efficacy of catnip oil as a natural mosquito repellent when used as the active ingredient in a lotion formulation and applied to human skin. The results show that lotion formulations containing 2% or 6% catnip oil were highly effective at repelling several different mosquito species from landing on human subjects under field conditions in rural Eastern Uganda. Moreover, 6% catnip oil lotion performed as well as the commercial mosquito repellent DEET and received very positive feedback from users in our acceptance trial. The relatively low concentration of catnip essential oil required for repellence means that the amount of catnip crop required for essential oil production is lower than for repellents requiring higher concentrations of the active ingredient. Hence, it may represent an effective, cheap, locally sourced and more sustainable alternative to DEET. This implies that, for people living in sub-Saharan Africa, catnip essential oil can provide protection for the period spent undertaking chores in the evening, when many species of mosquito are most active. The catnip plant is easily cultivated in much of sub-Saharan Africa and could be used as a nepetalactone feedstock for large-scale production of affordable mosquito repellents.

## Supplementary Information

Below is the link to the electronic supplementary material.

Supplementary Material 1

Supplementary Material 2

## Data Availability

The data generated in this study are available in the Supplementary Data spreadsheet.
