# Supplementary material for: Evaluating repellence properties of a catnip essential oil-based mosquito repellent using the human landing catch method in Eastern Uganda
Source: Sci Rep. 2026 Mar 14;16:13272. doi: 10.1038/s41598-026-42618-5 (PMC13106807; doi:10.1038/s41598-026-42618-5)
Supplement: Supplementary file 1 — Supplementary Material 1 [file 41598_2026_42618_MOESM1_ESM.pdf]

Supplementary data

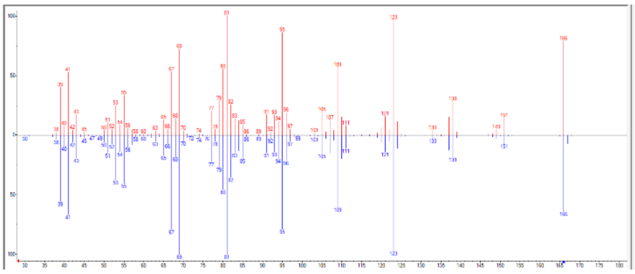

SSR-Nepetalactone

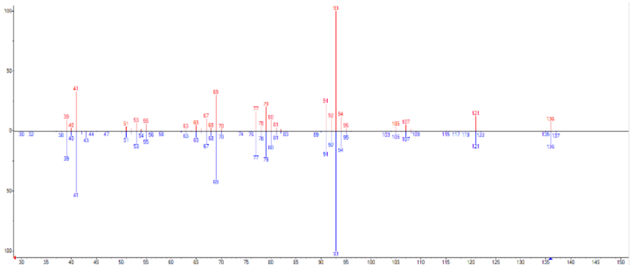

β-Pinene

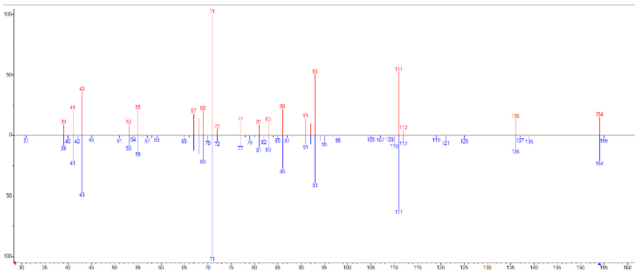

(-)-Terpinen-4-ol

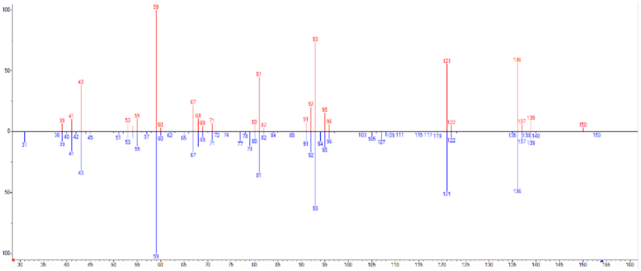

α-Terpineol

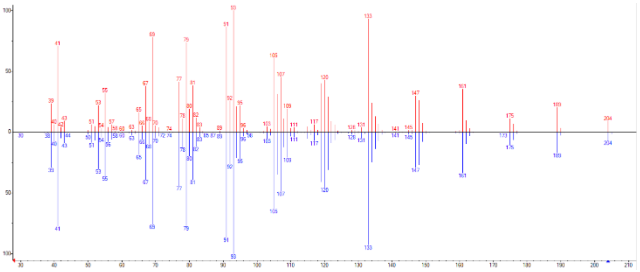

Caryophyllene

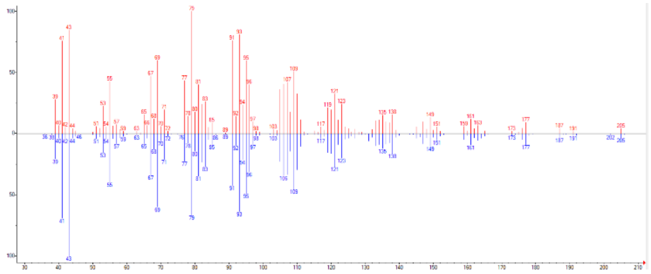

Caryophyllene oxide

Supplementary Figure S1. Head-to-tail spectral plots of measured (red) vs library (blue) mass spectra using NIST mass spectral library search version 2.2 for compounds identified in distilled catnip essential oil obtained by steam distillation of plant tissue from *Nepeta cataria* Chemotype A. Y-axis = relative abundance of each ion, X-axis = mass to charge ratio (m/z).
